# E-cigarette and cannabis use among current and recently quit smokers: Co-use and Co-cessation

**DOI:** 10.1016/j.abrep.2025.100611

**Published:** 2025-04-15

**Authors:** Deanna M. Halliday, Anna V. Song, Nhung Nguyen

**Affiliations:** a Center for Tobacco Control Research and Education, University of California, San Francisco, California, United States; b Department of Psychological Sciences, University of California, Merced, California, United States; c UC Merced’s Nicotine and Cannabis Policy Center, California, United States; d Division of General Internal Medicine, Department of Medicine, University of California, San Francisco, California, United States

**Keywords:** Tobacco, Cigarettes, E-cigarettes, Cannabis, Co-Use

## Abstract

**Background:**

Concurrent use of cigarettes with e-cigarette or cannabis (co-use) is common. It is unclear whether people who want to quit smoking cigarettes would also be interested in quitting using e-cigarettes/cannabis (co-cessation).

**Methods:**

In a survey of 391 Californian adults, participants reported past 30-day use of and intentions to quit cigarettes, e-cigarettes, and cannabis, and reasons for using e-cigarettes and/or cannabis. Using cross-tabulation tables, we examined the relationship between cigarette, e-cigarette, and cannabis cessation intentions. We subsequently examined how the reasons for using e-cigarettes and cannabis related to e-cigarette and cannabis use frequency, while controlling for cigarette use and demographic characteristics.

**Results:**

Of those who used both cigarettes and e-cigarettes and planned to quit smoking within the next 30 days, 68.9 % also planned to quit using e-cigarettes. Of those who used both cigarettes and cannabis and intended to quit smoking within 30 days, 30.0 % also wanted to quit using cannabis within the same period. Using e-cigarettes or cannabis to aid with cigarette cessation had no impact on e-cigarette or cannabis use frequency.

**Significance:**

Those who intended to quit smoking cigarettes within 30-days also expressed interest in quitting e-cigarettes or cannabis within the same period. Using e-cigarettes or cannabis to quit smoking was not related to higher frequency e-cigarette or cannabis use. Future interventions may promote co-cessation of cigarettes, e-cigarettes, and cannabis simultaneously.

## Highlights

• Co-use of cigarettes with e-cigarettes and cannabis is common.
• Among cigarette cessation seekers, many also want to quit e-cigs and cannabis.
• E-cigs or cannabis were likely not used to compensate for the loss of cigarettes.
• More frequent cannabis use was related to more frequent cigarette use.

## 1 Introduction

People who smoke cigarettes are more likely to use e-cigarettes and cannabis than those who do not smoke (Agrawal et al., 2012, Azagba, 2018, Khouja et al., 2021). Co-use (defined here as concurrently using cigarettes with e-cigarettes and/or cannabis) is more common than cigarette-only use (Reboussin et al., 2021, Wills et al., 2021). This is concerning given that co-use is associated with both independent and compounding health harms (Kim et al., 2020, Osei et al., 2019, Werner et al., 2020) and addiction (Morean et al., 2018, Rubinstein et al., 2014). There is little guidance about how to address co-use when supporting cigarette cessation, such as whether to concurrently promote e-cigarette/ cannabis cessation (co-cessation) or address cigarette cessation alone.

Those who smoke cigarettes use e-cigarettes and cannabis for many reasons, including as a cigarette cessation aid. Several randomized control trials have demonstrated the efficacy of e-cigarettes as a smoking cessation aid (Lindson et al., 2025), though evidence of the real-world effectiveness of e-cigarettes is mixed in population-based research (Al-Delaimy et al., 2015, Sweet et al., 2019, Wang et al., 2021, Weaver et al., 2018). However, continued e-cigarette use prolongs nicotine exposure and may increase nicotine consumption among those who successfully quit cigarettes and put those who fail to quit smoking at risk of long-term cigarette and e-cigarette co-use (Hanewinkel et al., 2022, Nguyen et al., 2023). Likewise, there is a strong correlation between nicotine and cannabis dependence (Hindocha and McClure, 2021, McClure et al., 2018), a relationship that is fostered both behaviorally through similar methods of consumption as well as biochemically through interactions between the nicotinic and endocannabinoid systems (Rabin & George, 2015). Previous research suggests that quitting both tobacco and cannabis improves the odds of successful tobacco cessation (Rogers et al., 2020), but interventions targeting cessation for both tobacco and cannabis are rare (Nguyen et al., 2024).

Previous research has focused on how e-cigarette or cannabis use impacts cigarette cessation, but less is known about how cigarette cessation-seekers perceive co-use and co-cessation. The majority of people who use e-cigarettes do not want to use these products long-term (Rosen & Steinberg, 2019), and some will struggle with quitting vaping much like those who smoke cigarettes struggle to quit smoking (Nguyen et al., 2023, Smith et al., 2021). However, there is little cessation support available for e-cigarette cessation and unassisted quitting is the most common (Struik & Yang, 2021). Likewise, the relationship between cigarette cessation and cannabis cessation has also been documented, yet not sufficiently addressed in cessation support services. Using cannabis is associated with lower odds of tobacco cessation (Driezen et al., 2021, Rogers et al., 2020), and tobacco use negatively affects successful cannabis cessation as well (McClure et al., 2018). One study found that among Quitline callers who used both cigarettes and cannabis, 43 % also wanted to reduce their cannabis consumption in addition to quitting smoking (Carpenter et al., 2020). Despite this interest in co-cessation for both tobacco and cannabis, there are few resources available.

There are two prevalent concerns regarding co-cessation: (1) it is unclear whether those who co-use would be interested in co-cessation (McClure et al., 2018) and (2) it is unknown to what extent cigarette cessation seekers may rely on e-cigarettes or cannabis to compensate for the loss of cigarettes. To address the first concern, this study aimed to describe how different stages of cigarette cessation intentions (i.e., no intention to quit, intention to quit in six months, and intention to quit in 30 days) relate to intentions to quit e-cigarettes and cannabis among co-users. As for the second concern, we examine if using e-cigarettes or cannabis for cigarette cessation is associated with higher frequency e-cigarette or cannabis use compared to those using e-cigarettes or cannabis for other reasons.

## 2 Materials and methods

### 2.1 Study design and sample

The survey was conducted in 2022 and 2023 both online via Prolific and in-person at community events (e.g., farmers’ markets, medical services fairs) in California’s San Joaquin Valley. Eligible participants were 18-years-old or older, proficient in English, and either currently smoking or recently quit smoking within 12 months. The sample included a total of 391 participants. All participants provided informed consent prior to participating in the survey and received incentives for completing the survey. At the in-person events, participants completed our survey on provided iPads and received a $10 gift card for completing the survey. Participants on Prolific received a $4.00 credit to their account, comparable to what is offered by other surveys. We used these multiple data collection methodologies to enhance our recruitment of participants from the less-populated regions of California. The study was approved by the University of California, Merced Institutional Review Board (UCM 2022–15).

### 2.2 Measures

*Demographics.* Participants reported age, gender (male, female, and gender minority), race/ethnicity, and educational attainment. Due to the small number of participants identifying as male-to-female transgender (n = 1), female-to-male transgender (n = 3), genderqueer (n = 4), and others (n = 1), we did not have the power to include these separate groups in the linear regression analyses. We have included a gender-minority category in Table 1 to represent these participants, however, in the regression model we use a binary male/female variable. For this, we recoded male-to-female transgender as female and female-to-male transgender to male, consistent with their gender identities.

**Table 1:** Sample Characteristics.

	Total Sample (n = 391)	Current Use of Cigarettes (n = 210)	Recently Quit Cigarettes (n = 181)	
	n(%) or mean (SD)	n(%) or mean (SD)	n(%) or mean (SD)	p-value
*Demographics*				
Age, M (SD)*	33.7 (11.0)	34.9 (11.8)	32.38 (9.8)	*p* = 0.061
Gender				*p* = 0.450
Male	225 (57.7 %)	122 (58.21 %)	103 (57.5 %)	
Female	155 (39.7 %)	85 (40.5 %)	70 (38.7 %)	
Gender Minority	10 (3.6 %)	3 (1.4 %)	6 (3.35)	
Race/ethnicity				*p* = 0.757
Non-Hispanic White	181 (46.3 %)	96 (45.7 %)	85 (47.0 %)	
Non-Hispanic Other	104 (26.6 %)	59 (28.1 %)	45 (24.9 %)	
Hispanic	106 (27.1 %)	55 (26.2 %)	51 (28.2 %)	
Education				*p* = 0.098
Less than 4-year degree	212 (54.2 %)	122 (58.1 %)	90 (49.7 %)	
4-year degree or more	179 (45.8 %)	88 (41.9 %)	91 (50.3 %)	

*Current Product Use*				
Uses Cigarettes	210 (53.7 %)	210 (100.0 %)	0 (0.0 %)	n/a
Uses E-cigarettes	169 (43.2 %)	115 (54.8 %)	54 (29.8 %)	***p <* 0.001**
Uses Cannabis	170 (43.5 %)	100 (47.6 %)	70 (36.7 %)	***p* = 0.040**
Uses E-cigarettes & Cannabis	93 (23.8 %)	62 (29.5 %)	31 (17.1 *%)*	***p* = 0.004**

*Product Use Average Days Per Month*				
Cigarettes M(SD)	16.4 (11.6)	16.4 (11.6)	0.0 (0.0)	n/a
E-cigarettes M(SD)*	17.9 (11.1)	17.1 (11.2)	19.4 (10.9)	*p* = 0.214
Cannabis M(SD)*	16.7 (11.3)	17.8 (11.4)	15.1 (11.1)	*p* = 0.135

**Note.** All p-values are the result of a chi-square test comparing current cigarette users to those recently quit with the exception of the analyses marked with an asterisk (*), which were assessed using Mann-Whitney U tests. Bolded values are significant.

*Use of cigarettes, e-cigarettes, and cannabis.* Participants reported on how many days of the past 30 days they used cigarettes, e-cigarettes, and cannabis as our measure of frequency of use. *Current use* of cigarettes was defined as having smoked more than 100 cigarettes in their lifetime and having smoked at least once in the past 30 days. *Recently quit* was defined as smoking more than 100 cigarettes lifetime, but not smoking cigarettes in the past 30 days. We further confirmed recently quit status by asking participants, “Are you a current or former smoker?” There were 15 participants who selected “current smoker” and were re-classified as current users of cigarettes regardless of their past 30-day use report.

If a participant indicated they had used e-cigarettes or cannabis in the past 30 days, they were considered currently using e-cigarettes or cannabis, respectively.

*Intention to quit each product.* Participants reported their intentions to quit separately for each of the products they used. They were asked to indicate if they were intending to quit each product in the next 30 days or six months, based on the Stages of Change model (DiClemente et al., 1991).

*Reasons for use.* Participants selected their primary reason for using e-cigarettes and cannabis from a provided list. For e-cigarettes, the reasons included to quit or cut down on smoking cigarettes, to use when they could not smoke, because they enjoy it, and because they were curious. For cannabis, the reasons included to quit or cut down on smoking cigarettes, because they enjoy it, because they were curious, and for medicinal purposes. Among those who had recently quit smoking cigarettes, primarily using e-cigarettes or cannabis to quit/reduce smoking cigarettes is interpreted as using these products to maintain smoking cessation. These reasons were then dichotomized to indicate that the products were either used to aid with cigarette cessation/cutting down on cigarettes or for any other reason.

### 2.3 Data analysis

All analyses were conducted using STATA 15.1. The Prolific online sample was significantly younger (M = 33.4, SD = 10.9) than the in-person samples (M = 39.6, SD = 11.2), but there were no other significant differences between the two samples. Given the similarity of the samples, we combined the Prolific and local samples in the analysis (See Table 1). Descriptive statistics of sample characteristics were summarized to compare those currently smoking (n = 210) and those who recently quit (n = 181). T-tests and Wilcoxon-Mann-Whitney tests were computed to examine differences between means of normally and non-normally distributed variables, respectively. Chi-square tests were used for categorical variables.

To examine the relationships between intentions to quit smoking cigarettes, e-cigarettes, and cannabis, we conducted Fisher’s exact tests due to small cell sizes. We compared intentions to quit e-cigarettes by intention to quit cigarettes among people who used both products (n = 115) and intentions to quit cannabis by intention to quit smoking cigarettes among people who used both products (n = 100), resulting in two 3x3 tables. We then calculated the adjusted standardized residuals (ASR) for each cell using the STATA command “tabchi” with the “adj” option. This allows us to determine which observed values deviated the most from the expected values, accounting for both the standard deviation of the expected value of the cell and the standard deviation of the residuals (Agresti, 2007). Because we are assessing nine cells, we used the Bonferroni adjustment to account for the number of tests, which reduced our significance level to 0.005. We use these analyses to identify the cells that deviate the most from the expected frequencies and determine whether that deviation is significant using standardized residuals to compute p-values.

We then ran separate linear regression models to determine if using e-cigarettes or cannabis to quit/reduce smoking was associated with frequency of e-cigarette use and cannabis use (two continuous outcomes) while controlling for demographic variables and cigarette use. To optimize the sample size for multivariate regression modeling, we chose to conduct two separate models with a larger sample size for each analysis (n = 169 for the model with e-cigarette use and n = 170 for the model with cannabis use). In these models, cigarette use was broken into a three-level categorical variable: former cigarette users, non-daily cigarette users, and daily cigarette users.

## 3 Results

### 3.1 Sample characteristics

Sample characteristics are reported in Table 1. The sample has an age range of 18–81, primarily identified as male (57.7 %) and non-Hispanic White (46.3 %). Of the total sample (n = 391), 169 currently used e-cigarettes and 170 currently used cannabis. Proportions of currently using e-cigarettes or cannabis were greater among those who were currently smoking cigarettes (54.8 % and 47.6 %, respectively) compared to those who had quit smoking cigarettes (29.8 % and 38.7 %, respectively). In the supplementary materials, Table A provides a detailed breakdown of differences in reasons for using e-cigarettes and cannabis as well as intentions to quit e-cigarettes and cannabis between those who currently smoke and those who had quit. Those who had recently quit smoking differed from those currently smoking with regards to their reasons to use e-cigarettes (*p* = 0.010) and their e-cigarette cessation intentions (*p* = 0.038).

### 3.2 Intentions to quit using Cigarettes, E-cigarettes, and cannabis

Among those who smoked cigarettes (n = 210), over half also used e-cigarettes (n = 115). Of those, 110 provided their intentions to quit cigarettes and e-cigarettes. Cigarette cessation intentions were common, with only 34 (30.9 %) reporting no intention to quit smoking, 31 (28.3 %) intending to quit within six months, and 45 (40.9 %) intending to quit within 30 days. Intentions to quit e-cigarettes were somewhat less common: 50 (45.5 %) reported no intention to quit, 23 (20.9 %) reported intentions to quit e-cigarettes within 6 months, and 37 (33.6 %) intended to quit within 30 days. To visualize the relationships between intentions to quit cigarettes and e-cigarettes, we calculated the percentage of those in the three e-cigarette cessation stages within those in the three cigarette cessation stages (See Fig. 1a). The majority (73.5 %) of those who had no intentions to quit smoking cigarettes (n = 34), also did not intend to quit e-cigarettes. Similarly, the majority (68.9 %) of those who intended to quit cigarettes within 30 days (n = 45) also intended to quit e-cigarettes in the same time period.

**Fig. 1a:**
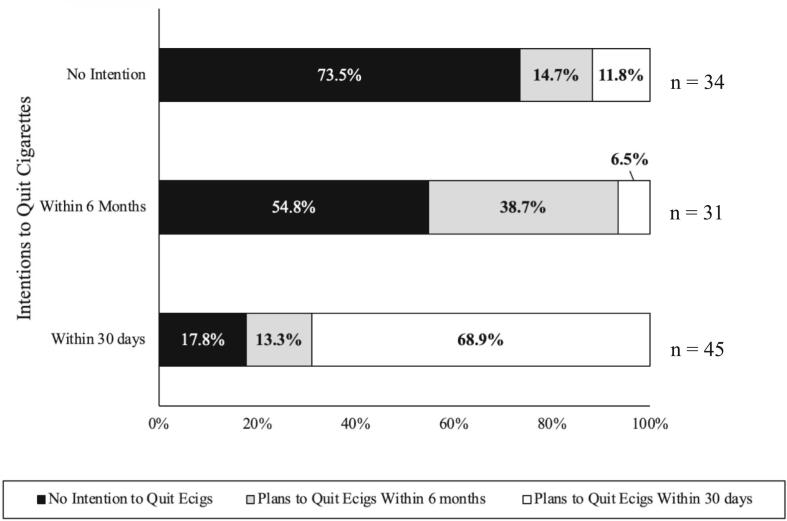
Intentions to Quit Cigarettes and E-cigarettes among Users of Both.

We conducted similar analyses among those who co-used cigarettes and cannabis (n = 100), of which 98 participants provided data. Again, more than half of the participants reported wanting to quit cigarettes: 33 (33.67) reported no intention to quit cigarettes, 25 (25.51 %) intended to quit within 6 months, and 40 (40.82 %) intended to quit within 30 days. Overall cannabis quit intentions were low, with 78 (79.59 %) reporting no intentions to quit cannabis, 5 (5.10 %) intending to quit within six months, and 15 (15.31 %) intending to quit within 30 days. Regardless, 30.0 % of those who planned to quit smoking cigarettes within 30 days (n = 40) also intended to quit using cannabis during the same time period (See Fig. 1b.).

**Fig. 1b:**
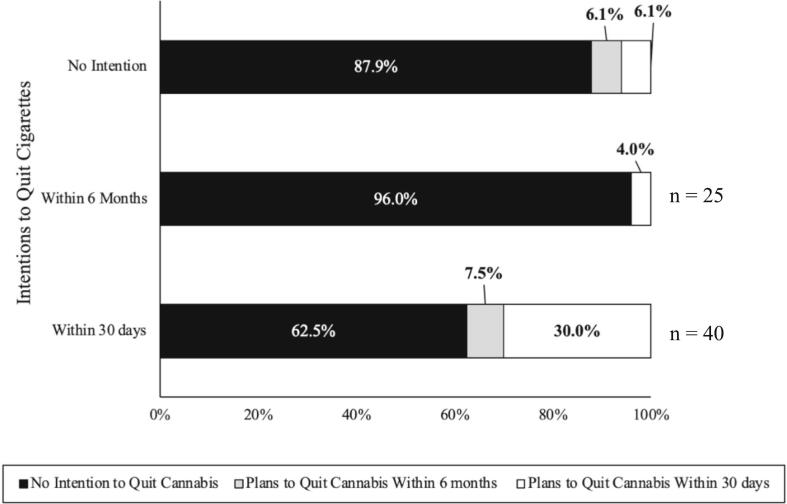
Intentions to Quit Cigarettes and Cannabis among Users of Both.

The relationship between cigarette and e-cigarette cessation intentions was significant (*p* < 0.001) (See Table 2a). We found that intentions to quit cigarettes and e-cigarettes aligned as those with no intentions to quit one product largely had no intention to quit the other, and those who wanted to quit one product in six months or 30 days were also likely to want to quit the other during the same period. The relationship between intentions to quit cigarettes and cannabis was also significant (*p* = 0.005) (See Table 2b), with intentions to quit cigarettes in the next 30 days relating to intentions to quit cannabis within 30 days.

**Table 2a:** Associations between Intentions to Quit Cigarettes and E-cigarettes among Users of Both.

		E-cigarette Cessation Intentions								
		No Quit Intention			Quit w/in 6 Months			Quit w/in 30 Days		
		n	ASR*	*p*	n	ASR*	*p*	n	ASR*	*p*
Cigarette Cessation Intentions n = 110	No Quit Intention	25	4.0	***p* < 0.001**	5	−1.1	*p* = 0.285	4	−3.2	***p* = 0.001**
	Quit w/in 6 Months	17	1.2	*p* = 0.216	12	2.9	***p* = 0.004**	2	−3.8	***p* < 0.001**
	Quit w/in 30 Days	8	−4.9	***p* < 0.001**	6	−1.6	*p* = 0.104	31	6.5	***p* < 0.001**

**Note:** p-value < 0.001 from Fisher's Exact Test; ASR: Adjusted Standard Residuals indicate the degree of difference between observed and expected values. We used the ASR to calculate an approximate p-value; n: number of participants; Bolded values are significant with the Bonferroni adjusted threshold of 0.005.

**Table 2b:** Associations between Intentions to Quit Cigarettes and Cannabis among Users of Both.

		Cannabis Cessation Intentions								
		No Quit Intention			Quit w/in 6 Months			Quit w/in 30 Days		
	n = 98	n	ASR*	*p*	n	ASR*	*p*	n	ASR*	*p*
Cigarette Cessation Intention n = 98	No Quit Intention	29	1.5	*p* = 0.147	2	0.3	*p* = 0.759	2	−1.8	*p* = 0.070
	Quit w/in 6 Months	24	2.4	*p* = 0.018	0	−1.3	*p* = 0.179	1	−1.8	*p* = 0.069
	Quit w/in 30 Days	25	−3.5	***p* < 0.001**	3	0.9	*p* = 0.370	12	2.4	***p* < 0.001**

**Note:** p-value < 0.001 from Fisher's Exact Test; ASR: Adjusted Standard Residuals indicate the degree of difference between observed and expected values. We used the ASR to calculate an approximate p-value; n: number of participants; Bolded values are significant with the Bonferroni adjusted threshold of 0.005.

### 3.3 Association between frequency of cigarette use with frequency of e-cigarette and cannabis use

Results from the linear regression models showed that those who were non-daily or daily cigarette users used e-cigarettes at a similar frequency as those who stopped using cigarettes. Those who were using e-cigarettes to aid with cigarette cessation did not differ in their e-cigarette use frequency compared to those using e-cigarettes for any other reason (*b* = 1.3; 95 %CI = −2.5, 5.1) (See Table 3). Notably, those who smoked cigarettes daily also used cannabis at a greater frequency compared to those who had quit smoking cigarettes (*b* = 6.0; 95 %CI = 0.7, 11.4). Those who did not smoke cigarettes daily did not differ from those who had quit smoking cigarettes in frequency of cannabis use. Again, frequency of cannabis use did not differ between those using cannabis to help them quit smoking compared to those using cannabis for any other reason (*b* = -3.3; 95 %CI = −10.0, 3.3). We checked the linearity assumptions for each model by examining the Residuals vs. Fitted Plot (rvfplot command in STATA) and found that residual patterns were scattered around zero, indicating the linearity assumptions held.

**Table 3:** Multivariate Linear Regression: Reasons for Product Use and Frequency of Use.

	Frequency of E-cigarette Use n = 169		Frequency of Cannabis Use n = 170	
	*b* (SE)	95 % CI	*b* (SE)	95 % CI
*Reasons for Product Use*				
Any Other Reason	ref.	ref.	ref.	ref.
To Quit or Reduce Cigarettes	1.3 (1.9)	−2.5 – 5.1	−3.3 (3.4)	−10.0 – 3.3

Frequency of Cigarette Use				
Recently Quit Smoking	ref.	ref.	ref.	ref.
Non-Daily Cigarette Use	−2.9 (2.0)	−6.8 – 1.1	1.1 (1.9)	−2.7 – 4.9
Daily Cigarette Use	2.8 (2.7)	−2.6 – 8.1	**6.0 (2.7)**	**0.7 – 11.4**

*Demographics*				
Age	0.1 (0.1)	−0.1 – 0.2	−0.1 (0.1)	−0.3 – 0.1
Gender				
Male	ref.	ref.	ref.	ref.
Female	−1.5 (1.8)	−5.1 – 2.1	1.4 (1.8)	−2.0 – 4.9

Race				
White Non-Hispanic	ref.	ref.	ref.	ref.
Non-white Non-Hispanic	1.8 (2.1)	−2.3 – 6.0	0.8 (2.1)	−3.4 – 4.9
Hispanic	3.3 (2.2)	−1.1 – 7.8	−1.0 (2.2)	−5.3 – 3.4

Education				
Less than 4-year degree	ref.	ref.	ref.	ref.
4-year degree or more	−0.7 (1.8)	−4.3 – 2.8	**−4.3 (1.8)**	**−7.8 – −0.7**

***Note.*** Bolded values are significant.

## 4 Discussion and Conclusions

This study examined associations between intentions to quit cigarettes, e-cigarettes, and cannabis. We also examined if e-cigarette or cannabis use frequency differed between those using those products for cigarette cessation compared to any other reason. This study addresses two common concerns about co-cessation interventions: 1) we demonstrate that there is an interest in co-cessation as we show intentions to quit using e-cigarettes or cannabis within 30 days were greatest among those who also planned to quit smoking cigarettes within 30 days, and 2) we did not see indications of a greater reliance on e-cigarettes or cannabis among those using these products to help them quit smoking cigarettes as they did not use e-cigarettes or cannabis more frequently compared to those using the products for other reasons. These findings support the pursuit of co-cessation interventions to help people quit these products simultaneously.

Determination for cessation aligned across the three products, showing that planning to quit using either e-cigarettes or cannabis was most common among those planning to quit smoking within 30 days. However, the relationships were notably stronger among those co-using cigarettes and e-cigarettes compared to those co-using cigarettes and cannabis. This may be due to the low number of participants who expressed a desire to quit using cannabis, however, 30-day quit intentions were still significantly related. While the stages of change (DiClemente et al., 1991) do not exactly align across the three products, the determination stage (e.g., intention to quit in 30 days) reflects those who are most prepared to make a quit attempt in the near future, a critical intervention point to provide support and encouragement (Armitage & Arden, 2008). Interest in co-cessation could be better assessed and supported among those determined to quit smoking cigarettes.

There is an unmet need for cessation support for those who want to quit multiple products and an unrealized potential to promote simultaneous cessation for cigarettes, e-cigarettes, and cannabis. Although some individuals may use e-cigarettes for cigarette cessation aids, evidence of the real-world effectiveness of e-cigarettes is inconclusive. A 2025 review of randomized control trials found moderate certainty that e-cigarettes outperform nicotine replacement therapy (Lindson et al., 2025), while another review showed limited effectiveness in population-based observational studies (Wang et al., 2021). It remains unclear whether consumer e-cigarettes used without clinical guidance improve the odds of cigarette cessation (Al-Delaimy et al., 2015, Sweet et al., 2019, Weaver et al., 2018). Because the degree to which e-cigarettes are beneficial for cigarette cessation is unclear, the impact of promoting co-cessation on long-term cigarette cessation is also unclear. A prolonged use of e-cigarettes after cigarette cessation may be an indicator that e-cigarettes are working as a replacement for cigarettes as intended (Butler et al., 2022). For those who find that e-cigarettes are the only way to prevent further smoking, co-use interventions would be counterproductive. However, it is also true that becoming a co-user of cigarettes and e-cigarettes is far more common than cessation of either product (Owusu et al., 2019) and co-use is associated with health harms (Kim et al., 2020, Osei et al., 2019). With the significant interest in co-cessation, further research is needed to provide guidance to those who want to quit one or both products to maximize the odds of quitting and minimize the odds of prolonged co-use.

There should also be greater awareness and greater encouragement to quit cannabis alongside tobacco. Overall desire to quit cannabis was low in our sample, but it is unclear whether participants are aware that their continued use of cannabis may impact successful tobacco cessation. A common concern in smoking cessation for tobacco and cannabis co-users is the risk of substituting or increasing cannabis use to compensate for reduced nicotine intake. However, our findings indicate that those using e-cigarettes or cannabis for cessation are not using these products at a higher rate than those using them for other purposes. This is consistent with a longitudinal study that indicated that a reduction in tobacco use is linked to a reduction, not an increase, in cannabis use (Nguyen et al., 2022). A recent review further provides support for well-integrated multi-component interventions of behavioral and pharmacotherapy strategies for co-cessation of tobacco and cannabis (Nguyen et al., 2024). This review also calls for research to determine the most effective treatment strategies addressing various product combinations and different co-use patterns, such as the combined use of cannabis and tobacco in combustible/smoking products.

This study has several limitations. First, this cross-sectional design cannot determine whether intentions to quit cigarettes cause intentions to quit e-cigarettes or cannabis or whether using cannabis or e-cigarettes to quit/reduce smoking resulted in a change in the frequency of using e-cigarettes or cannabis. Second, our measure of the current use of each product may not capture people who were during the quitting process, and it is unclear if our findings would be robust if more stringent definitions were used. Additionally, the small sample size recruited in California and the convenience sampling procedure limit study generalization. Data on co-use patterns of tobacco and cannabis and factors associated with desires for co-cessation (e.g., duration of use of each product and tobacco and cannabis dependency) were not collected.

This study challenges the previously held concerns about the co-cessation of cigarettes, e-cigarettes, and cannabis by showing the positive correlations between intention to quit these products and similar frequency of e-cigarette or cannabis use among those using these products for cessation compared to those using the products for other reasons. Our findings call for the development of interventions that allow those who currently smoke to capitalize on their smoking cessation efforts and achieve cessation of e-cigarettes and cannabis should they desire it.

## CRediT authorship contribution statement

**Deanna M. Halliday:** Writing – original draft, Visualization, Project administration, Methodology, Investigation, Funding acquisition, Formal analysis, Data curation, Conceptualization. **Anna V. Song:** Writing – review & editing, Supervision, Resources, Methodology, Funding acquisition. **Nhung Nguyen:** Writing – review & editing, Supervision.

## Funding

Funding was provided by the Tobacco-Related Disease Research Program (TRDRP) pre-doctoral fellowship award (T32DT4828) and the TRDRP grant number 28PC-0044. The funders had no input on the study design, collection, analysis or interpretation of the data, writing the manuscript, or the decision to submit the paper for publication

## Declaration of competing interest

The authors declare that they have no known competing financial interests or personal relationships that could have appeared to influence the work reported in this paper.

## Data Availability

Data will be made available on request.

## References

[b0005] Agrawal A., Budney A.J., Lynskey M.T. (2012). The co-occurring use and misuse of cannabis and tobacco: A review. Addiction.

[b0010] Agresti A. (2007).

[b0015] Al-Delaimy W.K., Myers M.G., Leas E.C., Strong D.R., Hofstetter C.R. (2015). E-cigarette use in the past and quitting behavior in the future: A population-based study. American Journal of Public Health.

[b0020] Armitage C.J., Arden M.A. (2008). How useful are the stages of change for targeting interventions? Randomized test of a brief intervention to reduce smoking. Health Psychology.

[b0025] Azagba S. (2018). E-cigarette use, dual use of e-cigarettes and tobacco cigarettes, and frequency of cannabis use among high school students. Addictive Behaviors.

[b0030] Butler A.R., Lindson N., Fanshawe T.R., Theodoulou A., Begh R., Hajek P., McRobbie H., Bullen C., Notley C., Rigotti N.A., Hartmann-Boyce J. (2022). Longer-term use of electronic cigarettes when provided as a stop smoking aid: Systematic review with meta-analyses. Preventive Medicine.

[b0035] Carpenter K.M., Torres A.J., Salmon E.E., Carlini B.H., Vickerman K.A., Schauer G.L., Bush T. (2020). Marijuana Use and adherence to smoking cessation treatment among callers to tobacco quitlines. Preventing Chronic Disease.

[b0040] DiClemente C.C., Prochaska J.O., Fairhurst S.K., Velicer W.F., Velasquez M.M., Rossi J.S. (1991). The process of smoking cessation: An analysis of precontemplation, contemplation, and preparation stages of change. Journal of Consulting and Clinical Psychology.

[b0045] Driezen P., Gravely S., Wadsworth E., Smith D.M., Loewen R., Hammond D., Li L., Abramovici H., McNeill A., Borland R., Cummings K.M. (2022). Increasing cannabis use is associated with poorer cigarette smoking cessation outcomes: Findings from the ITC four country smoking and vaping surveys, 2016–2018. Nicotine & Tobacco Research.

[b0050] Hanewinkel R., Niederberger K., Pedersen A., Unger J.B., Galimov A. (2022). E-cigarettes and nicotine abstinence: A meta-analysis of randomised controlled trials. European Respiratory Review.

[b0055] Hindocha C., McClure E.A. (2021). Unknown population-level harms of cannabis and tobacco co-use: If you don’t measure it, you can’t manage it. Addiction.

[b0060] Khouja J.N., Suddell S.F., Peters S.E., Taylor A.E., Munafò M.R. (2021). Is e-cigarette use in non-smoking young adults associated with later smoking? A systematic review and meta-analysis. Tobacco Control.

[b0065] Kim C.-Y., Paek Y.-J., Seo H.G., Cheong Y.S., Lee C.M., Park S.M., Park D.W., Lee K. (2020). Dual use of electronic and conventional cigarettes is associated with higher cardiovascular risk factors in Korean men. Scientific Reports.

[b0070] Lindson N., Butler A.R., McRobbie H., Bullen C., Hajek P., Wu A.D., Begh R., Theodoulou A., Notley C., Rigotti N.A., Turner T., Livingstone-Banks J., Morris T., Hartmann-Boyce J. (2025). Electronic cigarettes for smoking cessation—Lindson, N - 2025 | Cochrane Library. Cochrane Database of Systematic Reviews.

[b0075] McClure E.A., Baker N.L., Sonne S.C., Ghitza U.E., Tomko R.L., Montgomery L., Babalonis S., Terry G.E., Gray K.M. (2018). Tobacco use during cannabis cessation: Use patterns and impact on abstinence in a national drug abuse treatment clinical trials network study. Drug and Alcohol Dependence.

[b0080] Morean M., Krishnan-Sarin S., O’Malley S.S. (2018). Comparing cigarette and e-cigarette dependence and predicting frequency of smoking and e-cigarette use in dual-users of cigarettes and e-cigarettes. Addictive Behaviors.

[b0085] Nguyen N., Bold K.W., McClure E.A. (2024). Urgent need for treatment addressing co-use of tobacco and cannabis: An updated review and considerations for future interventions. Addictive Behaviors.

[b0090] Nguyen N., Koester K.A., Kim M., Watkins S.L., Ling P.M. (2023). “I’m both smoking and vaping”: A longitudinal qualitative study of US young adults who tried to quit smoking cigarettes by using electronic cigarettes. Tobacco Control.

[b0095] Nguyen N., Neilands T.B., Lisha N.E., Lyu J.C., Olson S.S., Ling P.M. (2022). Longitudinal associations between use of tobacco and cannabis among people who smoke cigarettes in real-world smoking cessation treatment. Journal of Addiction Medicine.

[b0100] Osei A.D., Mirbolouk M., Orimoloye O.A., Dzaye O., Uddin S.M.I., Benjamin E.J., Hall M.E., DeFilippis A.P., Stokes A., Bhatnagar A., Nasir K., Blaha M.J. (2019). Association between E-cigarette use and cardiovascular disease among never and current combustible-cigarette smokers. The American Journal of Medicine.

[b0105] Owusu D., Huang J., Weaver S.R., Pechacek T.F., Ashley D.L., Nayak P., Eriksen M.P. (2019). Patterns and trends of dual use of e-cigarettes and cigarettes among US adults, 2015–2018. Preventive Medicine Reports.

[b0110] Rabin R.A., George T.P. (2015). A review of co-morbid tobacco and cannabis use disorders: Possible mechanisms to explain high rates of co-use. The American Journal on Addictions.

[b0115] Reboussin B.A., Wagoner K.G., Ross J.C., Suerken C.K., Sutfin E.L. (2021). Tobacco and marijuana co-use in a cohort of young adults: Patterns, correlates and reasons for co-use. Drug and Alcohol Dependence.

[b0120] Rogers A.H., Shepherd J.M., Buckner J.D., Garey L., Manning K., Orr M.F., Schmidt N.B., Zvolensky M.J. (2020). Current cannabis use and smoking cessation among treatment seeking combustible smokers. Drug and Alcohol Dependence.

[b0125] Rosen R.L., Steinberg M.L. (2019). Interest in quitting E-cigarettes among adults in the United States. Nicotine & Tobacco Research.

[b0130] Rubinstein M.L., Rait M.A., Prochaska J.J. (2014). Frequent marijuana use is associated with greater nicotine addiction in adolescent smokers. Drug and Alcohol Dependence.

[b0135] Smith T.T., Nahhas G.J., Carpenter M.J., Squeglia L.M., Diaz V.A., Leventhal A.M., Dahne J. (2021). Intention to quit vaping among United States adolescents. JAMA Pediatrics.

[b0140] Struik L., Yang Y. (2021). E-cigarette cessation: content analysis of a quit vaping community on reddit. Journal of Medical Internet Research.

[b0145] Sweet L., Brasky T.M., Cooper S., Doogan N., Hinton A., Klein E.G., Nagaraja H., Quisenberry A., Xi W., Wewers M.E. (2019). Quitting behaviors among dual cigarette and E-cigarette users and cigarette smokers enrolled in the tobacco user adult cohort. Nicotine & Tobacco Research: Official Journal of the Society for Research on Nicotine and Tobacco.

[b0150] Wang R.J., Bhadriraju S., Glantz S.A. (2021). E-cigarette use and adult cigarette smoking cessation: A meta-analysis. American Journal of Public Health.

[b0155] Weaver S.R., Huang J., Pechacek T.F., Heath J.W., Ashley D.L., Eriksen M.P. (2018). Are electronic nicotine delivery systems helping cigarette smokers quit? Evidence from a prospective cohort study of US adult smokers, 2015–2016. PloS one.

[b0160] Werner A.K., Koumans E.H., Chatham-Stephens K., Salvatore P.P., Armatas C., Byers P., Clark C.R., Ghinai I., Holzbauer S.M., Navarette K.A., Danielson M.L., Ellington S., Moritz E.D., Petersen E.E., Kiernan E.A., Baldwin G.T., Briss P., Jones C.M., King B.A., Reagan-Steiner S. (2020). Hospitalizations and deaths associated with EVALI. New England Journal of Medicine.

[b0165] Wills T.A., Leventhal A.M., Sargent J.D., Pagano I. (2021). Concurrent use of E-cigarettes, combustible cigarettes, and marijuana. Pediatrics.

