# Supplementary material for: E-cigarette and cannabis use among current and recently quit smokers: Co-use and Co-cessation
Source: Addict Behav Rep. 2025 Apr 15;21:100611. doi: 10.1016/j.abrep.2025.100611 (PMC12033952; doi:10.1016/j.abrep.2025.100611)
Supplement: Supplementary Data 1 [file mmc1.docx]

Supplementary Material

**Table A.**

*Detailed Product Use Characteristics by Cigarette Use Status*

|  | | Total Sample  (n = 391) | Current Use of Cigarettes  (n = 210) | Recently Quit Cigarettes  (n = 181) |  |
| --- | --- | --- | --- | --- | --- |
| *Reasons for Product Use* | n(%) | | n(%) | n(%) | p-value |
| E-cigarettes | *n = 169* | | *n = 115* | *n = 54* | ***p =* 0.010** |
| To quit smoking | 83 (49.1%) | | 29 (24.4%) | 30 (55.6%) |  |
| To cut down on smoking | 45 (26.6%) | | 35 (30.7%) | 10 (18.5%) |  |
| To use when I cannot smoke | 10 (5.9%) | | 7 (6.1%) | 3 (5.6%) |  |
| Because I enjoy it | 49 (29.9%) | | 39 (34.2%) | 10 (18.5%) |  |
| Curiosity | 4 (2.4%) | | 3 (2.6%) | 1 (1.9%) |  |
| Some other reason | 1 (0.6%) | | 1 (0.9%) | 0 (0.0%) |  |
| Cannabis | *n = 170* | | *n = 100* | *n = 70* |  |
| To quit smoking | 8 (4.7%) | | 4 (4.0%) | 4 (5.7%) | *p* = 1.90 |
| To cut down on smoking | 4 (2.4%) | | 4 (4.0%) | 0 (0.0%) |  |
| Medical reasons | 47 (27.6%) | | 29 (29.0%) | 18 (25.7%) |  |
| Because I enjoy it | 109 (64.1%) | | 63 (63.0%) | 46 (65.7%) |  |
| Curiosity | 2 (1.2%) | | 0 (0.0%) | 2 (2.9%) |  |
| *Intentions to Quit* |  | |  |  |  |
| Cigarettes | *n = 210* | | *n = 210* | *n = 0* | n/a |
| No intention to quit | 70 (33.3%) | | 70 (33.3%) | - |  |
| Quit w/in 6 months | 50 (23.8%) | | 50 (23.8%) | - |  |
| Quit w/in 30 days | 86 (41.0%) | | 86 (41.0%) | - |  |
| E-cigarettes | *n = 169* | | *n = 115* | *n = 54* | ***p =* 0.038** |
| No intention to quit | 64 (37.9%) | | 51 (44.3%) | 13 (24.1%) |  |
| Quit w/in 6 months | 42 (24.9%) | | 25 (21.7%) | 17 (31.5%) |  |
| Quit w/in 30 days | 60 (35.5%) | | 37 (32.2%) | 23 (42.6%) |  |
| Cannabis | *n = 170* | | *n = 100* | *n = 70* | *p =* .161 |
| No intention to quit | 136 (80.0%) | | 79 (79.0%) | 57 (81.4%) |  |
| Quit w/in 6 months | 12 (7.1%) | | 5 (5.0%) | 7 (10.0%) |  |
| Quit w/in 30 days | 20 (11.8%) | | 15 (15.0%) | 5 (7.1%) |  |

**Note:** All p-values are the result of a chi-square test. Italicized n’s represent the number of participants in a subgroup of the overall n (i.e., there are 169 e-cigarette users in the total sample, 115 among current users of cigarettes, and 54 among those who have recently quit) as described in the methods.
